# Supplementary material for: Root-Associated Microbiomes of Panax notoginseng under the Combined Effect of Plant Development and Alpinia officinarum Hance Essential Oil
Source: Molecules. 2022 Sep 15;27(18):6014. doi: 10.3390/molecules27186014 (PMC9501277; doi:10.3390/molecules27186014)
Supplement: Supplementary file 1 [file molecules-27-06014-s001.zip › molecules-1901135-supplementary.pdf]

Supplementary materials

A.

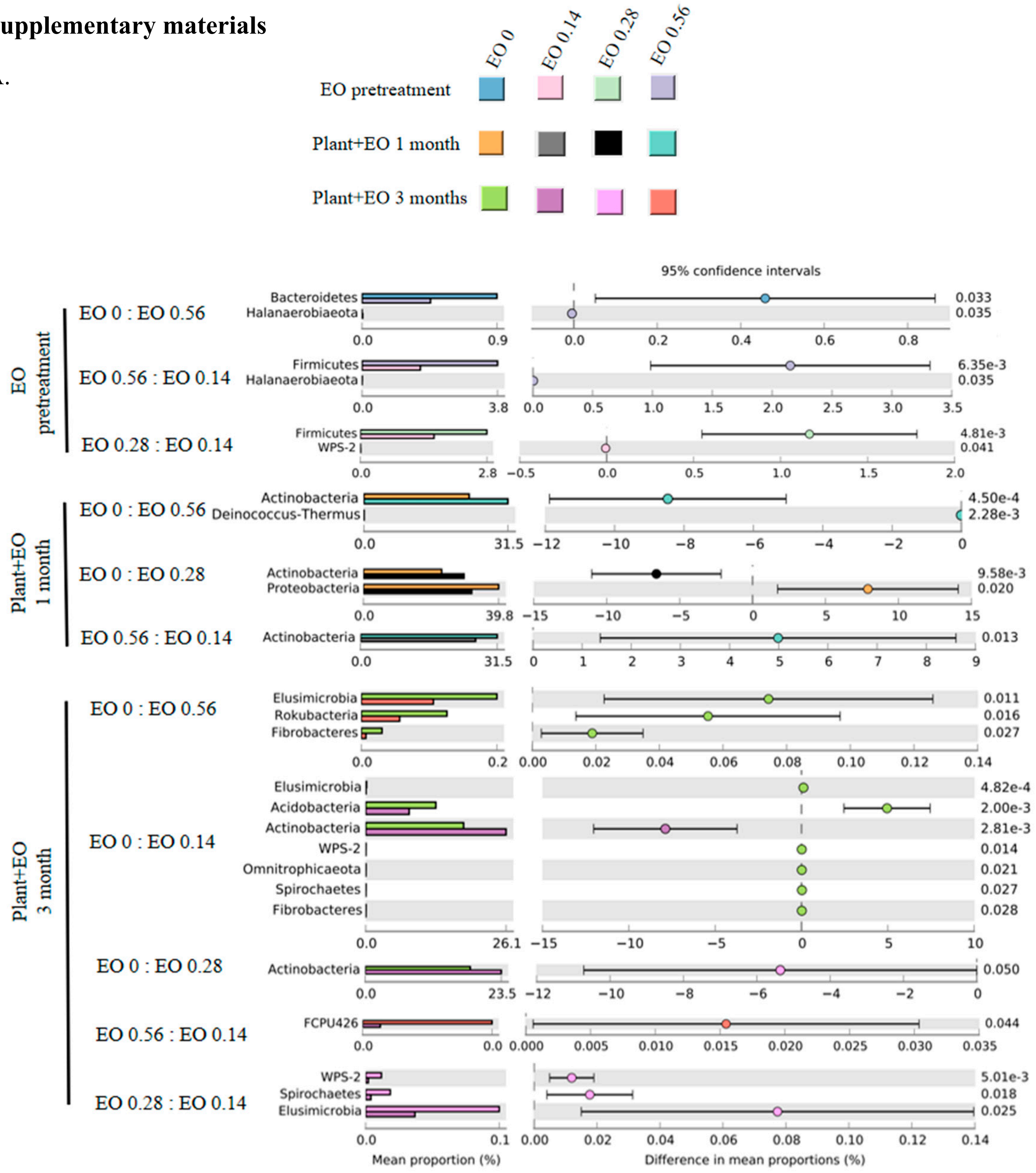

B.

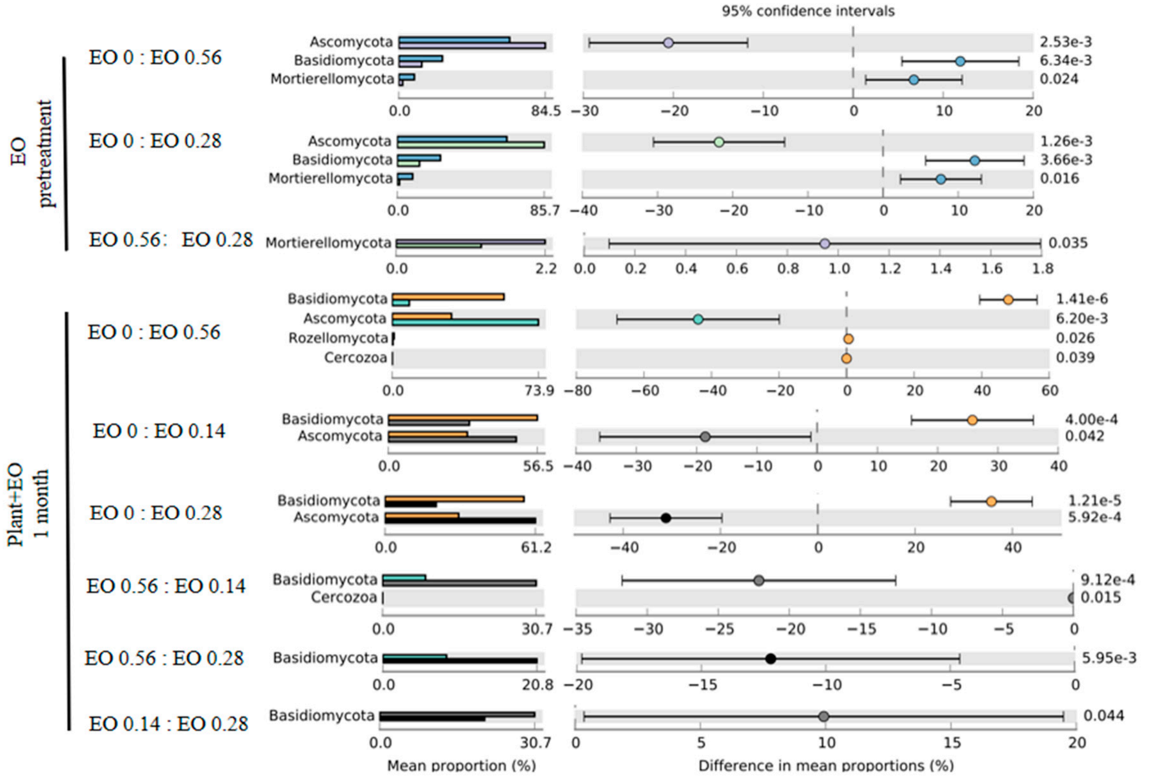

Figure S1. The pure effect of EOs and the combined effect of EOs and *P. notoginseng* growth on bacteria (**A**) and fungal (**B**) community composition after 1 month and 3 months. The concentrations of EO in the soil were 0 mg/g (control), 0.14 mg/g (low concentration), 0.28 mg/g (medium concentration) and 0.56 mg/g (high concentration). The three stages were: EO pretreatment for 7 days, then *P. notoginseng* was planted and samples were assessed at 1 and 3 months.

A.

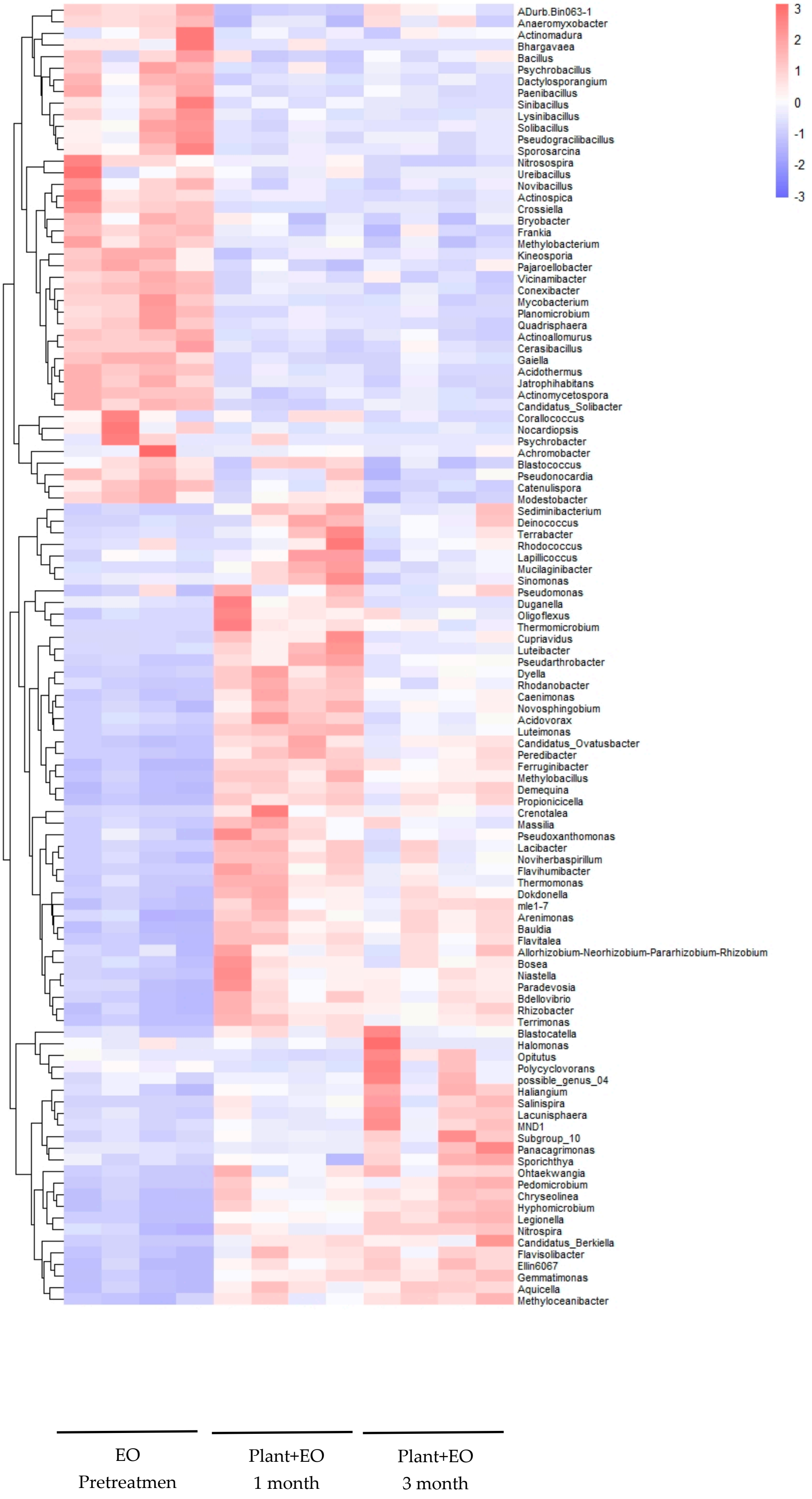
3
2
1
0
-1
-2
-3
ADurb.Bin063-1
Anaeromyxobacter
Actinomadura
Bhargavaea
Bacillus
Psychrobacillus
Dactylosporangium
Paenibacillus
Sinibacillus
Lysinibacillus
Solibacillus
Pseudogracilibacillus
Sporosarcina
Nitrosospira
Ureibacillus
Novibacillus
Actinospica
Crossiella
Bryobacter
Frankia
Methylobacterium
Kineosporia
Pajaroellobacter
Vicinamibacter
Conexibacter
Mycobacterium
Planomicrobium
Quadrisphaera
Actinoallomurus
Cerasibacillus
Gaiella
Acidothermus
Jatrophihabitans
Actinomycetospora
Candidatus_Solibacter
Corallococcus
Nocardiopsis
Psychrobacter
Achromobacter
Blastococcus
Pseudonocardia
Catenulispora
Modestobacter
Sediminibacterium
Deinococcus
Terrabacter
Rhodococcus
Lapillicoccus
Mucilaginibacter
Sinomonas
Pseudomonas
Duganella
Oligoflexus
Thermomicrobium
Cupriavidus
Luteibacter
Pseudarthrobacter
Dyella
Rhodanobacter
Caenimonas
Novosphingobium
Acidovorax
Luteimonas
Candidatus_Ovatusbacter
Peredibacter
Ferruginibacter
Methylobacillus
Demequina
Propionicicella
Crenotalea
Massilia
Pseudoxanthomonas
Lacibacter
Noviherbaspirillum
Flavihumibacter
Thermomonas
Dokdonella
mle1-7
Arenimonas
Bauldia
Flavitalea
Allorhizobium-Neorhizobium-Pararhizobium-Rhizobium
Bosea
Niastella
Paradevosia
Bdellovibrio
Rhizobacter
Terrimonas
Blastocatella
Halomonas
Opitutus
Polycyclovorans
possible_genus_04
Haliangium
Salinispira
Lacunisphaera
MND1
Subgroup_10
Panacagrimonas
Sporichthya
Ohtaekwangia
Pedomicrobium
Chryseolinea
Hyphomicrobium
Legionella
Nitrospira
Candidatus_Berkiella
Flavisolibacter
Ellin6067
Gemmatimonas
Aquicella
Methyloceanibacter
EO
Pretreatmen
Plant+EO
1 month
Plant+EO
3 month

B.

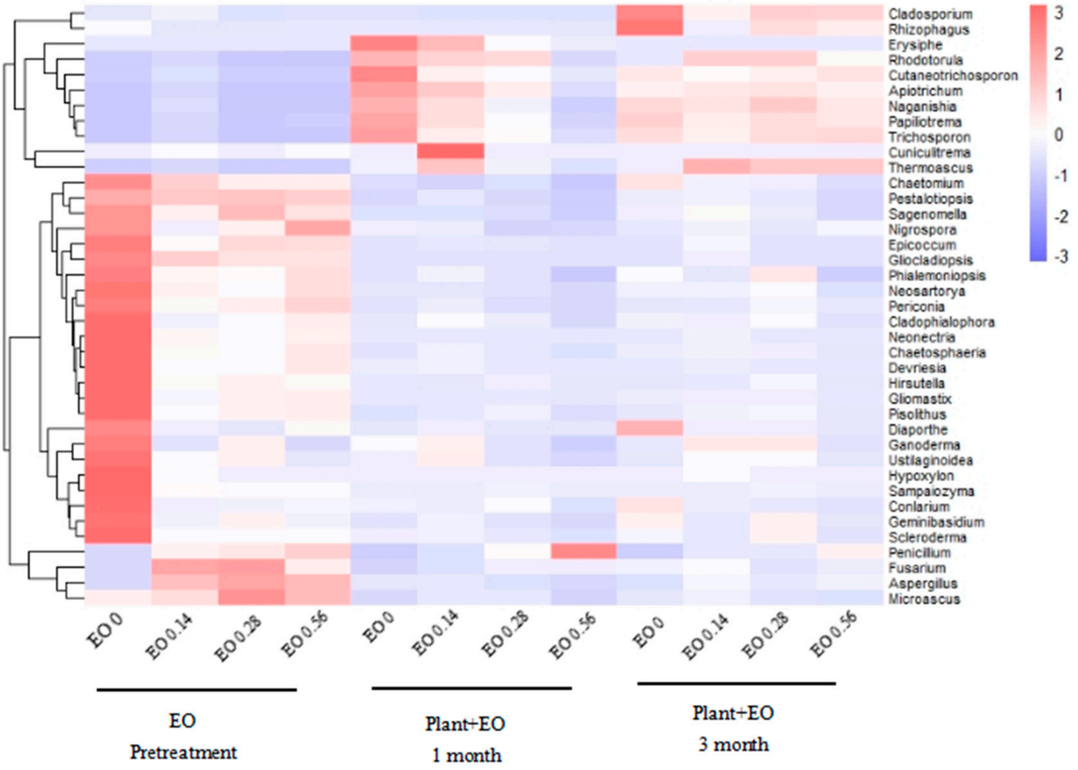

Figure S2. Bacterial (**A**) and fungal (**B**) community composition of the rhizosphere soil samples at the genus level. The concentrations of EO in the soil were 0 mg/g (control), 0.14 mg/g (low concentration), 0.28 mg/g (medium concentration) and 0.56 mg/g (high concentration). The three stages were: EO pretreatment for 7 days, then *P. notoginseng* was planted and samples were assessed at 1 and 3 months.

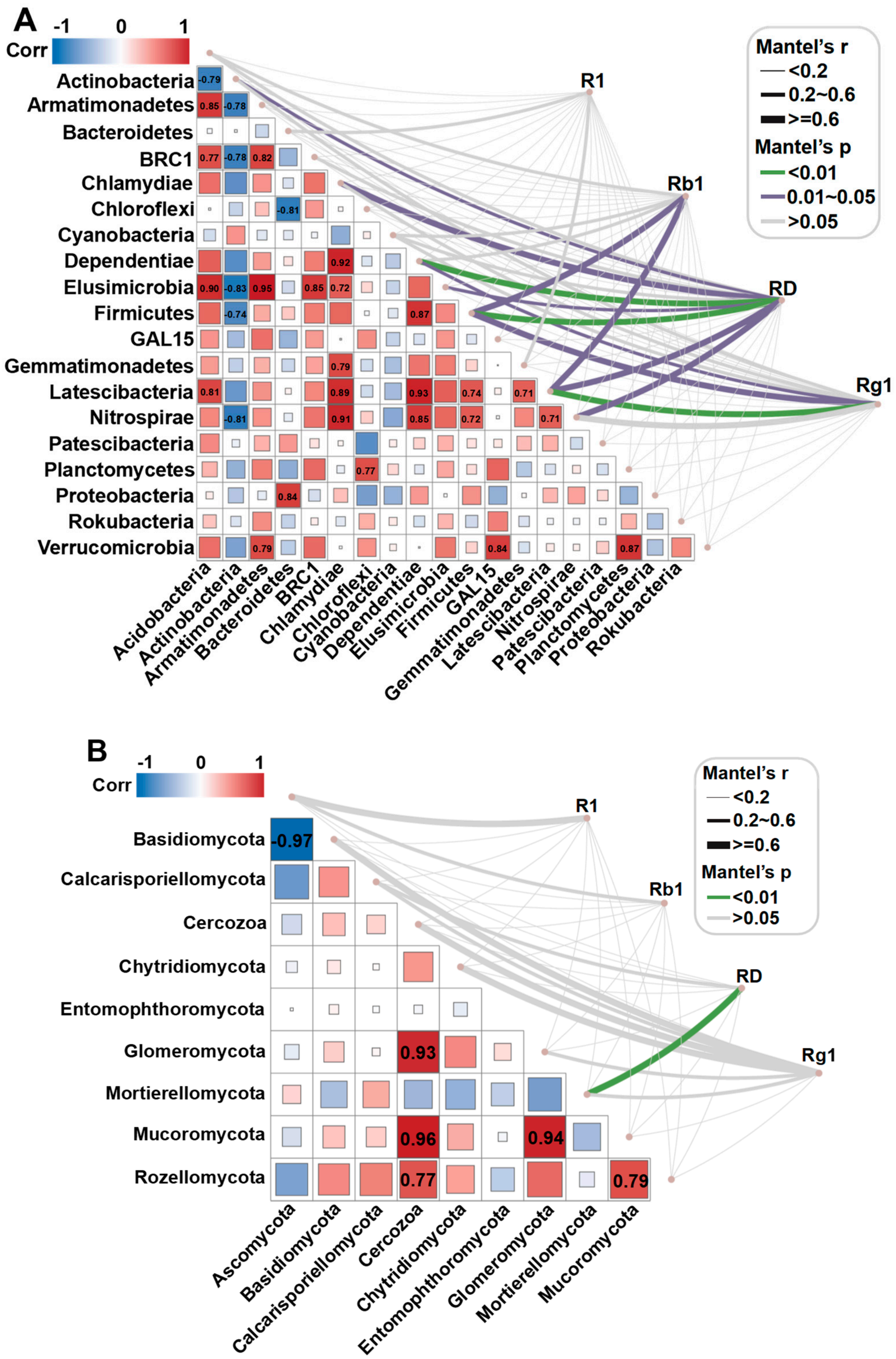

Figure S3. Pairwise comparisons of Phylum-level distribution of the 16S (**A**) and ITS (**B**) root microbiota are shown, with a color gradient denoting pearson's correlation coefficients. Saponin content (R1, Rb1, Rd

and Rg1) (B) were correlated with the species of the community by mantel test. Edge width corresponds to the Mantel's r statistic for the corresponding distance correlations, and edge color denotes the statistical significance.
